# Peering Strategic Game Models for Interdependent ISPs in Content Centric Internet

**DOI:** 10.1155/2013/380265

**Published:** 2013-12-05

**Authors:** Jia Zhao, Jianfeng Guan, Changqiao Xu, Wei Su, Hongke Zhang

**Affiliations:** ^1^National Engineering Laboratory for Next Generation Internet Interconnection Devices, Beijing Jiaotong University, Beijing 100044, China; ^2^State Key Laboratory of Networking and Switching Technology, Beijing University of Posts and Telecommunications, Beijing, China; ^3^Institute of Sensing Technology and Business, Beijing University of Posts and Telecommunications, Wuxi, Jiangsu, China

## Abstract

Emergent content-oriented networks prompt Internet service providers (ISPs) to evolve and take major responsibility for content delivery. Numerous content items and varying content popularities motivate interdependence between peering ISPs to elaborate their content caching and sharing strategies. In this paper, we propose the concept of peering for content exchange between interdependent ISPs in content centric Internet to minimize content delivery cost by a proper peering strategy. We model four peering strategic games to formulate four types of peering relationships between ISPs who are characterized by varying degrees of cooperative willingness from egoism to altruism and interconnected as profit-individuals or profit-coalition. Simulation results show the price of anarchy (PoA) and communication cost in the four games to validate that ISPs should decide their peering strategies by balancing intradomain content demand and interdomain peering relations for an optimal cost of content delivery.

## 1. Introduction

Tremendous volume of traffic from content-oriented services such as media streaming and file download motivates the evolution of Internet architecture for more efficient content delivery. With the emergence of new networking paradigms such as content-centric networking (CCN) [1], the design of future Internet trends towards the way to take content as a central entity. In such content-centric Internet, Internet service providers (ISPs) attach storage to their distributed network nodes (e.g., routers) for in-network caching and delivering content locally [2]. Such extended caching function prompts ISPs in the future Internet to evolve from traffic managers to content managers that will take the major responsibility for content delivery [3, 4].

Caching and delivering content by ISPs give rise to the question as to what kind of interrelationship ISPs can build to fulfill quality of service (QoS) for their intradomain content requesters. Unlike the traditional interconnection (through peering or transit contracts) to maintain global reachability in the current Internet, description of interrelations between ISPs in content centric Internet is more complex. This is so because achieving QoS of content delivery is a tough work that calls for interdependence between ISPs. Although an ISP can cache its intradomain popular content, there are numerous content items whose heavy tail of popularity distribution [5] and varying content popularity across different networks make it difficult for the ISP to satisfy intradomain content requests independently with its limited cache capacity. Accordingly, cooperative caching or inter-ISP content sharing is a more reasonable way to a win-win situation for ISPs [5]. Traditional ISP interconnection through traffic transit or peering should be supplemented with an interdependent relationship (i.e., peering for content) between ISPs so that the communication cost of content delivery is optimized.

**Figure 1:**
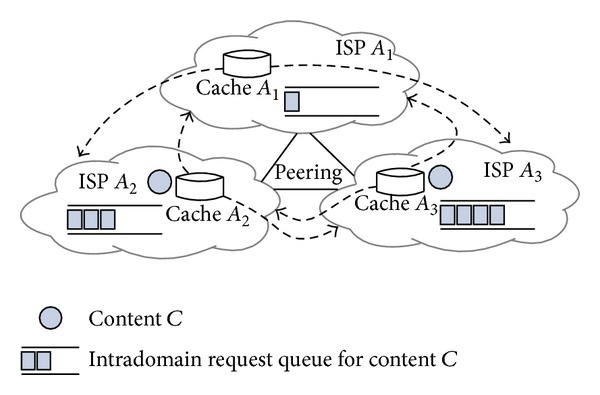
Interdependent ISPs *A*_1_, *A*_2_, and *A*_3_ build peering relationships between each other through cache-to-cache content sharing. Request queues for content *C* within the three ISPs indicate intradomain popularity of content *C*. Both *A*_2_ and *A*_3_ cache the content *C*. *A*_1_ does not cache *C* but can receive it from *A*_2_ or *A*_3_.

A topology at autonomous system (AS) level consists of multiple ISPs with selfish profit utility and variety of complicated bilateral or multilateral relationships. So, it is difficult to let all ISPs converge to a consensus of cooperation. According to intradomain content popularity and profit goal for optimal communication cost (e.g., content delivery distance or latency and cache updating overhead), an ISP should utilize flexible and applicable caching and sharing strategies to build peering relationships with other ISPs. Peering ISPs can make full use of their content caching and sharing flexibility and commit themselves to deliver their intradomain requested content at low cost. For example, in Figure 1, requests for content *C* in ISP *A*_2_ or *A*_3_ are much more than the requests in *A*_1_. *A*_2_ and *A*_3_ cache the popular content *C* to lower cross-ISP delivery cost, while *A*_1_ can receive content transit from *A*_2_ or *A*_3_ to serve its less popular content demand without caching the content. If the peering is based on reciprocity, both *A*_2_ and *A*_3_ have the right to decide how they will contribute according to *A*_1_'s contribution. If *A*_1_, *A*_2_ and *A*_3_ are three sub-ISPs organized as coalition by a larger ISP, the dominant ISP can coordinate the caches and adjust sharing mechanism for coalitional profit maximization. Variety of peering strategies is due to two aspects: (i) the degree of an ISP's cooperative willingness varying from egoism to altruism, (ii) profit relationships such as profit-individual ISPs or a profit-coalition of ISPs.

In this paper, we model four peering strategic games with interdependent ISPs as participants, each of which decides its caching strategy (i.e., whether to cache a content item) and sharing strategy (i.e., how many interdomain content requests to respond to) to minimize the communication cost of content delivery. In the egoistic game, each ISP can decide its own caching strategy and respond to all its peering ISP's content requests. In the tit-for-tat game, an ISP decides its sharing strategy according to its peer's sharing strategy so as to achieve the reciprocity. In the altruistic game, two peeing ISPs embody a common profit goal in their respective cost function to save cost of content exchange between each other. In the cooperative game, ISPs cooperate as a coalition to gain more profit than noncoalitional ISPs. These four types of interdependent patterns are modeled to describe possible relationships between peering ISPs in content-centric Internet. In subsequent sections, we base our problem analysis and propositions in this paper on the circumstance where ISPs are equipped with the content-centric in-network cache and responsible for content delivery. Some conclusions are also well suited to ISP-operated content delivery networks (CDNs) and Internet economics (e.g., peering or transit) happening between ISPs in the current Internet. We simulate the four games based on an AS-level topology representing the interconnection between some large networks of the Chinaese Internet. Performance evaluation shows that comparatively altruistic games (altruism and cooperation) manifest agreeable properties (low PoA and cost) under high frequency of content requests and that sharing strategy also affects delivery cost in the tit-for-Tat case. This means that ISPs should decide their peering strategies by balancing intradomain content demand and interdomain peering relations for an optimal cost of content delivery.

The main contributions of this paper includes the following: (1) we propose the concept of peering for content exchange between interdependent ISPs in the content centric Internet to minimize their respective content delivery cost by proper peering strategies; (2) we use four game models to formulate and describe the interdependence between peering ISPs; (3) we study the peering ISPs' communication cost of content delivery and each game's divergence from social optimum to validate that ISPs should decide their peering strategies by balancing intradomain content demand and interdomain peering relation.

The remainder of the paper is organized as follow.  Section 2 introduces related work. In Section 3, we detail the peering strategy and give the cost utility expression. In Section 4, we model the four peering strategic games.  Section 5 makes some game-theoretic analysis. Simulation results are shown in Section 6.  Section 7 discusses some involving issues.  Section 8 concludes the paper.

## 2. Related Work

Peering strategy for inter-ISP content sharing is essential to both the current and the future Internet. With the Internet evolution from hierarchical to flat structure, settlement-free peering prevails among ISPs and prompts reciprocal traffic exchange between interconnected ISPs. Widespread video content distribution and its tremendous traffic volume motivate the peering between CDN providers and ISPs. In the paper [6], the authors investigate the content peering between ISPs and large content providers and develop a model to probe into the interaction between different types of ISPs. The authors demonstrate that the situation of asymmetric traffic from and into the CDN providers may not benefit the ISPs and violate the basic peering principle of reciprocity.

Content distributed systems such as CDN and P2P pose significant challenge on the traffic peering or transit relationship between ISPs [7, 8]. ISPs deploy intradomain content cache so as to decrease inter-ISP traffic. In the paper [5], the author formulates two game models to illustrate that ISPs can cooperate to improve selfish interests with cooperative caching strategies. For the efficiency of content delivery such as P2P streaming, collaborative caching policy of peering ISPs is also proposed to save content-receiving cost [9].

ISP interconnectivity in the current Internet experiences change and adjustment. In [10], the authors build an agent-based network model to study interdomain ecosystem and demonstrate evolutionary Internet transition from a transit hierarchy to a peering mesh. Varying peering or transit strategies are proposed and implemented according to ISP's selfish profit utility and relationship with each other, such as the work in [11].

In the current Internet, ISPs and content distributed system operators share a common profit goal to respond to content requests as locally as possible. Our proposed peering by cooperative caching strategy can satisfy intradomain content demand at optimal content-receiving cost and alleviate the situation of asymmetric profits in [6]. Also differing from the work in [5, 9], due to an ISP's extended duty to deliver content, our proposed peering strategic game models gain equilibrium solution to the optimization of an ISP's content delivery utility (i.e., intradomain contentreceiving cost measured by distance or latency). Additionally, intended to elaborate peering strategy for inter-ISP content sharing, our work also supplements, diversifies and evolves the peering patterns of ISPs.

## 3. Preliminaries

### 3.1. From Interconnection to Interdependence

Based on the principle of global reachability in the current Internet, interconnected ISPs commit themselves to agreements or contracts to deal with peering traffic or transit traffic. As the Internet architecture evolves to be content-oriented, ISPs will actively participate in the content delivery more than just traffic management. ISPs will act as CDN operators or cache content in their distributed network nodes so as to satisfy intradomain content demand. Content centric Internet motivates the peering relationship changed from interconnection to interdependence for more efficient content delivery. Peering ISPs can take advantage of the cross-ISP content delivery (if the content requester is geographically far away from its intradomain cache but nearby the content source of another ISP), and the huge global cache capacity can favor every ISP and enhance its respective caching flexibility (i.e., caching the popular by itself and requesting the less popular from other peers). Peering for content calls for solutions to select proper caching and sharing strategy, which is the focus of our work.

### 3.2. Peering Strategies

Let Φ = {*A*_1_,…, *A*_*m*_} represent a set of peering ISPs. For all *A*_*i*_ ∈ Φ, ISP *A*_*i*_ has a total cache capacity *C*(*A*_*i*_). Here we consider the distributed cache of an ISP as a whole, because every node (without generality, regarded as a router) with storage for content caching function belongs to the ISP's network whose cache function is independent of the other peering ISPs.

Γ = {*c*_0_, *c*_1_,…*c*_*n*_} represents the content item set, *S*(*c*_*j*_) is the size of content *c*_*j*_ (also applicable to the case of total size of multiple copies of *c*_*j*_ within an ISP), and *P*(*c*_*j*_) denotes the global popularity of content *c*_*j*_. Content popularity can reflect the request frequency for a content item. Yet, in the real life of applications such as P2P systems, content popularity distribution is of the heavy tail [5]. This means that the less popular content items are too numerous to omit. We conform to this pattern and set *P*(*c*_*j*_) to follow a Zipf-like distribution [12] in our simulation. With this content popularity over multiple ISPs, we can assign ISP *A*_*i*_ a part of *P*(*c*_*j*_) to denote the intradomain content popularity *p*_*A*_*i*__^(*c*_*j*_)^ which is subject to ∑_*A*_*i*__ *p*_*A*_*i*__^(*c*_*j*_)^ = *P*(*c*_*j*_).

Given the previous denotations, for the peering relationship between ISPs, each ISP can independently decide two peering strategies as follows.

- Caching strategy: *I*_*A*_*i*__^(*c*_*j*_)^ denotes the caching strategic function getting value 1 if ISP *A*_*i*_ caches the content *c*_*j*_ and value 0 otherwise. The decision *I*_*A*_*i*__^(*c*_*j*_)^ of ISP *A*_*i*_ is influenced by both the cache capacity constraint of ∑_*c*_*j*__ *I*_*A*_*i*__^(*c*_*j*_)^ · *S*(*c*_*j*_) ≤ *C*(*A*_*i*_) and the intradomain content popularity *p*_*A*_*i*__^(*c*_*j*_)^.
- Sharing strategy: *X*_*ik*_^(*c*_*j*_)^ ∈ [0,1] is a fraction of *p*_*A*_*k*__^(*c*_*j*_)^ and denotes how much the ISP *A*_*i*_ is sharing to respond to ISP *A*_*k*_'s requests for content *c*_*j*_. Because of the contradiction between the limited cache capacity and the content popularity in heavy tail distribution, ISPs receive the shared content from each other for their respective intradomain less popular requests and build an interdependent relationship on this sharing mechanism. How much sharing depends on, however, the patterns of peering is we will discuss in the next section. After all, the sharing is also the basis of our proposed peering for the content centric Internet.

### 3.3. QoS Metric and Utility Function

We employ game models to study the peering patterns. Every peering ISP, as a participant in a game, has a utility function to optimize its own profit objective. In the content centric Internet, peering ISPs are supposed to deliver content efficiently for their respective intradomain requesters. So, we set the objective to optimize a QoS metric of content delivery.

To define the QoS metric, we have to select a metric of cost to deliver content. Let *D*_*A*_*i*__^(*c*_*j*_)^ denote the cost of ISP *A*_*i*_ to deliver the content *c*_*j*_, and the QoS metric of ISP *A*_*i*_ can be formulated as follows:

(1) $$\begin{array}{c} U_{A_{i}}=\sum_{c_{j}}p_{A_{i}}^{\left(c_{j}\right)}\cdot D_{A_{i}}^{\left(c_{j}\right)}, \end{array}$$

where *U*_*A*_*i*__ is the total cost as the utility. *D*_*A*_*i*__^(*c*_*j*_)^ depends on the strategies of peering ISPs. An ISP decides its caching and sharing strategies to minimize its QoS metric. In application, *D*_*A*_*i*__^(*c*_*j*_)^ has its meaning at distinct grain-levels as follows.

- Coarse-grained: *D*_*A*_*i*__^(*c*_*j*_)^ denotes the AS-level hop-count distance from the responder ISP to the requester ISP *A*_*i*_.
- Fine-grained: *D*_*A*_*i*__^(*c*_*j*_)^ denotes the average delay or distance that the requester ISP *A*_*i*_ cost to receive content *c*_*j*_ within itself or from the responder ISPs.

Formula (1) with a simple form can calculate the QoS metric with the fine-grained denotation of *D*_*A*_*i*__^(*c*_*j*_)^, but it cannot show the ISP's peering strategies *I*_*A*_*i*__^(*c*_*j*_)^and *X*_*ik*_^(*c*_*j*_)^ obviously. Accordingly, we use a composition of the two definitions of *D*_*A*_*i*__^(*c*_*j*_)^. Let *d*_*A*_*i*__^(*c*_*j*_)^ denote the content delivery cost (intradomain content-delivering distance or delay) within an ISP *A*_*i*_, *q*_*A*_*i*__^(*c*_*j*_)^ denote the cross-ISP communication cost to receive content from a neighboring ISP (one AS-hop away), and *r*_*A*_*i*__^(*c*_*j*_)^ denote the cost to receive content from a remote ISP (multi-AS-hop away) or directly from content providers (CPs). Then the utility of ISP *A*_*i*_ can be expressed as follows:

(2) UAi=∑cjpAi(cj)·(dAi(cj)·IAi(cj)+(1−IAi(cj))·qAi(cj)·∑k≠iIAk(cj)·Xki(cj)        + rAi(cj)·(1−IAi(cj))·(1−∑k≠iIAk(cj)·Xki(cj))),

with constraint of ∑_*k*≠*i*_ *I*_*A*_*k*__^(*c*_*j*_)^ · *X*_*ki*_^(*c*_*j*_)^ ≤ 1. The formulating part as ∑_*k*≠*i*_ *I*_*A*_*k*__^(*c*_*j*_)^ · *X*_*ki*_^(*c*_*j*_)^ means the condition that the peering ISPs cache the content *c*_*j*_ and contribute to ISP *A*_*i*_ with their respective sharing strategies. *q*_*A*_*i*__^(*c*_*j*_)^ is normally greater than *d*_*A*_*i*__^(*c*_*j*_)^, but when the content requester is geographically far away from its intradomain cache but nearby the content source of another ISP, we have *q*_*A*_*i*__^(*c*_*j*_)^ < *d*_*A*_*i*__^(*c*_*j*_)^. In Section 4, we use the utility of form like (2) to model the peering strategic games. In the simulation, for simplicity without generality, we use the AS-hop-count to evaluate the content delivery cost.

### 3.4. Number of Game Participants

Peering for traffic in the current Internet is traditionally built between two same tier level ISPs (though there is also the peering between two different levels of ISPs). In the content centric Internet, ISPs have various patterns of interdependent relationship (which will be seen in the next section). Because of the variety of peering for content and an ISP's dependent choice of peering strategy, the complex games may exist among multiple participants that play with each other directly or indirectly. Yet, some of these complex games (e.g., egoism, tit-for-tat, and altruism in the next section) can be generated by a series of noncooperative games between a pair of ISPs. So, we will use the basic two-player game to describe these peering patterns. There are still, however, some peering patterns (e.g., cooperation) involving multiple participants, and we employ cooperative or noncooperative games of multiple players to deal with these cases.

## 4. Interdependence Patterns

In this section, interdependence patterns of ISPs are formulated as the peering strategic games. An ISP caches popular content for in-network content delivery, while it can also request content from its peer ISP if it does not cache the content, or this cross-ISP content delivery unfolds efficiency (e.g., content source belonging to ISP *A*_1_ is geographically located nearby the requester in ISP *A*_2_).

### 4.1. Egoism

This peering is built between two profit-individual ISPs in content centric Internet. Either ISP is egoistic to optimize its own content delivery utility. A noncooperative game [13] with ISP *A*_1_ and *A*_2_ as two players can be modeled as follows

*A*_1_ decides *I*_*A*_1__^(*c*_*j*_)^ to minimize:

(3) UA1=∑cjpA1(cj)·(dA1(cj)·IA1(cj)+qA1(cj)·(1−IA1(cj))·IA2(cj)       + rA1(cj)·(1−IA1(cj))·(1−IA2(cj))),

subject to ∑_*c*_*j*__ *I*_*A*_1__^(*c*_*j*_)^·*S*(*c*_*j*_) ≤ *C*(*A*_1_).

*A*_2_ decides *I*_*A*_2__^(*c*_*j*_)^ to minimize

(4) UA2=∑cjpA2(cj)·(dA2(cj)·IA2(cj)+qA2(cj)·(1−IA2(cj))·IA1(cj)       + rA2(cj)·(1−IA1(cj))·(1−IA2(cj))),

subject to ∑_*c*_*j*__ *I*_*A*_2__^(*c*_*j*_)^·*S*(*c*_*j*_) ≤ *C*(*A*_2_).

Both the ISPs decide their caching strategies selfishly and independently, and their profit goals to minimize their respective cost of content delivery converge them to a strategic equilibrium (i.e., the best caching strategy, whose existence will be proved in next section). To reduce latency or distance of content delivery, egoistic ISPs have to respond to content requests of each other, since there may be *q*_*A*_*i*__^(*c*_*j*_)^ < *d*_*A*_*i*__^(*c*_*j*_)^. The free exchange of content (with sharing strategy as *X*_12_^(*c*_*j*_)^ = *X*_21_^(*c*_*j*_)^ = 1) in this game is similar to the settlement-free agreement between peering ISPs in the current Internet to exchange traffic by transit-free. Hence, the egoistic peering for content can be built between two ISPs at a same tier level because of similar number of clients and cache capacities.

### 4.2. Tit-for-Tat

In the “tit-for-tat,” ISPs *A*_1_ and *A*_2_, *A*_1_ will decide its sharing strategy *X*_12_^(*c*_*j*_)^ according to the *A*_2_'s decision *X*_21_^(*c*_*j*_)^ and vice versa. A noncooperative game with ISPs *A*_1_ and *A*_2_ as two players can be modeled as follows:

*A*_1_ decides *I*_*A*_1__^(*c*_*j*_)^ and *f*_1_(*X*_21_^(*c*_*j*_)^) to minimize

(5) UA1=∑cjpA1(cj)·(dA1(cj)·IA1(cj)+qA1(cj)·f2(X12(cj))·(1−IA1(cj))·IA2(cj)         + rA1(cj)·(1−IA1(cj))·(1−X21(cj)·IA2(cj))),

subject to ∑_*c*_*j*__ *I*_*A*_1__^(*c*_*j*_)^·*S*(*c*_*j*_) ≤ *C*(*A*_1_), 0 ≤ *f*_1_(*X*_21_^(*c*_*j*_)^) ≤ 1.

*A*_2_ decides *I*_*A*_2__^(*c*_*j*_)^ and *f*_2_(*X*_12_^(*c*_*j*_)^) to minimize

(6) UA2=∑cjpA2(cj)·(dA2(cj)·IA2(cj)+qA2(cj)·f1(X21(cj))·(1−IA2(cj))·IA1(cj)        + rA2(cj)·(1−IA2(cj))·(1−X12(cj)·IA1(cj))),

subject to ∑_*c*_*j*__ *I*_*A*_2__^(*c*_*j*_)^·*S*(*c*_*j*_) ≤ *C*(*A*_2_), 0 ≤ *f*_2_(*X*_12_^(*c*_*j*_)^) ≤ 1.

We use function *f*_1_(*X*_21_^(*c*_*j*_)^) to express *X*_12_^(*c*_*j*_)^ not only because we want to show the reciprocity in “tit-for-tat,” but also we will analyze the type and influence of sharing strategies in the simulations. An ISP in this game can partially respond to content requests from the other and build the peering on the base of reciprocity. In the content sharing, the content requester ISP only optimizes its utility to reduce content delivery delay or distance for quality of service, while the responder ISP has to take the expense of content transit. Hence, differing from free exchange in the egoism, “tit-for-tat” allows an ISP to relate its own sharing strategies with the other ISP's contribution. An example is the equational exchange formulated as *X*_12_^(*c*_*j*_)^ = *f*_1_(*X*_21_^(*c*_*j*_)^) = *f*_2_(*X*_12_^(*c*_*j*_)^) = *X*_21_^(*c*_*j*_)^.

### 4.3. Altruism

Altruistic ISPs can join in a coalition. The utility of a coalition member embodies not only the selfish utility as formula (3) or (4) but also a coalition cost as follows:

(7) UA1,A2=∑cj(pA1(cj)+pA2(cj))·(D·(IA1(cj)∪IA2(cj))            +D′·(IA1(cj)∪IA2(cj)¯)),

where *D* denotes the content-receiving cost within the coalition and *D*′ denotes the cost to receive content from an ISP out of the coalition. A game between ISP *A*_1_ and *A*_2_ is modeled as follows:

*A*_1_ decides *I*_*A*_1__^(*c*_*j*_)^ to solve the problem as follows:

(8) $$\begin{array}{c} \mathrm{Min}\;U_{A_{1}}+U_{A_{1},A_{2}}, \end{array}$$

subject to ∑_*c*_*j*__ *I*_*A*_1__^(*c*_*j*_)^·*S*(*c*_*j*_) ≤ *C*(*A*_1_).

*A*_2_ decides *I*_*A*_2__^(*c*_*j*_)^ to solve the problem as follows:

(9) $$\begin{array}{c} \mathrm{Min}\;U_{A_{2}}+U_{A_{1},A_{2}}, \end{array}$$

subject to ∑_*c*_*j*__ *I*_*A*_2__^(*c*_*j*_)^·*S*(*c*_*j*_) ≤ *C*(*A*_2_).

The altruistic peering is applicable to the relation between multiple ISPs that agree to be a coalition and share the cost of intracoalitional content delivery. Although the ISPs serve their respective clients, this peering agreement allows the members of the coalition to pursue an optimal coalitional utility without losing their selfish profit. To understand the necessity of this altruism case, we see a similar example where two public peering ISPs buy bandwidth from Internet exchange point (IXP) operators or build private link between each other.

### 4.4. Cooperation

Altruistic coalition in Section 4.3 is a noncooperative game between two selfish ISPs although they embody a coalition cost utility in their own utility functions. In this subsection, we propose another type of coalition that can be modeled by a cooperative game [14]. A cooperative game differs from a noncooperative game in that participants in the coalition pursue a higher profit than situation where they do not participate in the coalition or cooperate with other ones out of the coalition.

**Proposition 1**

Two ISPs *A*_1_ and *A*_2_ with same content demand (*p*_*A*_1__^(*c*_*j*_)^ = *p*_*A*_2__^(*c*_*j*_)^) can cooperate to form a stable coalition to save content delivery cost.

**Proof**

*A*_1_, *A*_2_, and *A*_3_ are three ISPs. Let {*c*_1_, *c*_2_, *c*_3_} denote the set of content items. *A*_1_ caches the content *c*_1_, *A*_2_ caches the content *c*_2_, and *A*_3_ caches the content *c*_3_. The advantage of the coalition lies in that cross-ISP content-receiving cost *D*_1_ equals intra-ISP cost *D*_0_ within the coalition. The three ISPs have same content demand (*p*_*A*_1__^(*c*_*j*_)^ = *p*_*A*_2__^(*c*_*j*_)^ = *p*_*A*_3__^(*c*_*j*_)^ = 1/3). If *A*_1_ and *A*_2_ cooperate to be a coalition {*A*_1_, *A*_2_}, a cooperative game is formed as follows.

According to formula (1), we get cost utility of *A*_1_ in the coalition as

(10) UA1  {A1,A2}=∑cjpA1(cj)·(D0·IA1(cj)+D0·(1−IA1(cj))·IA2(cj)       + D1·(1−IA1(cj))·(1−IA2(cj))·IA3(cj))=23·D0+13·D1.

If the two ISPs do not cooperate, the utility of *A*_1_ is as follows.

(11) UA1  {A1}=∑cjpA1(cj)·(D0·IA1(cj)+D1·(1−IA1(cj))·IA2(cj)        + D1·(1−IA1(cj))·(1−IA2(cj))·IA3(cj))=13·D0+23·D1.

Because *D*_1_ > *D*_0_, we have *U*_*A*_2__^_{*A*_1_,*A*_2_}_^ = *U*_*A*_1__^_{*A*_1_,*A*_2_}_^ < *U*_*A*_2__^_{*A*_2_}_^ = *U*_*A*_1__^_{*A*_1_}_^. Either *A*_1_ or *A*_2_ in other coalitions such as {*A*_1_, *A*_3_} or {*A*_2_, *A*_3_} will not gain more profit. So, *A*_1_ and *A*_2_ can cooperate to be a stable coalition for cost saving.

An ISP may be in charge of multiple ASs and arrange content-caching to organize the ASs as a profit-coalition. Let *p*_AS_*i*__^(*c*_*j*_)^ denote the content demand of *c*_*j*_ within AS_*i*_ and *D*_AS_*i*__^(*c*_*j*_)^ denote AS_*i*_'s cost to deliver content *c*_*j*_. The dominant ISP assigns each AS a caching strategy to optimize coalitional content delivery cost by solving the following problem:

(12) $$\begin{array}{c} \mathrm{Min}\;U_{\text{Coalition}}=\sum_{c_{j}}\sum_{{\text{AS}}_{i}}p_{{\text{AS}}_{i}}^{\left(c_{j}\right)}\cdot D_{{\text{AS}}_{i}}^{\left(c_{j}\right)}. \end{array}$$

In this optimization, ASs in the coalition cooperate to achieve a common profit goal.

### 4.5. Global Optimum

Global optimum is an ideal state in which all the ISPs decide their caching strategy by optimizing a global profit utility together. Let *D*_*A*_*i*__(*x*) denote the ISP *A*_*i*_'s cost to deliver content *x* and *p*_*A*_*i*__(*x*) denote the content demand (i.e., content popular density) of content *x* within ISP *A*_*i*_. The global popular density of content *x* is expressed as *p*(*x*) = ∑_*A*_*i*__ *p*_*A*_*i*__(*x*). Because *p*(*x*) follows a Zipf distribution, we have ∫_*c*_0__^*c*_*n*_^ *p*(*x*) · d*x* = 1. Here, *c*_*o*_ and *c*_*n*_, respectively, denote the most and the least popular content. The utility of ISP *A*_*i*_ is expressed as *U*_*A*_*i*__(*x*) = *p*_*A*_*i*__(*x*) · *D*_*A*_*i*__(*x*). ISPs decide their caching strategies by solving the problem as follows:

(13) $$\begin{array}{c} \mathrm{Min}\;U_{\text{Global}}=\int_{c_{0}}^{c_{n}}\left(\sum_{A_{i}}U_{A_{i}}\left(x\right)\right)\cdot \text{d}x, \end{array}$$

subject to ∫_*c*_0__^*c*_*n*_^ *I*_*A*_*i*__(*x*) · *S*(*x*) · d*x* ≤ *C*(*A*_*i*_).

In practice, the global AS-level topology has a multitude of ASs operated by multiple ISPs that pursue their optimal selfish profit. Although the global optimum exists, the caching strategy at the global optimum is not adopted by ISPs. Yet, if an equilibrium solution exists in a peering strategic game, the global optimum can be used to evaluate different game's optimum cost divergences from the social optimum so as to maintain a global efficiency of content delivery.

## 5. Game-Theoretic Analysis

We have built the game models to study the interdependence between peering ISPs, and some following questions arise: (1) whether the equilibrium solutions exist in these games? (2) How can the peering ISPs as participants converge to the equilibrium in the actual game process? (3) What properties can be used to evaluate the equilibrium. This section will respond to these questions.

### 5.1. Existence of Equilibria

Existence of the equilibrium point in the game of egoism case is firstly investigated with the following proposition.

**Proposition 2**

A Nash equilibrium solution exists in the noncooperative game with two egoistic ISPs as participants.

**Proof**

Formulas (3) and (4) are the utility functions of two egoistic ISPs *A*_1_ and *A*_2_. Let two sets *I*_1_ = {*I*_*A*_1__^(*c*_0_)^,…, *I*_*A*_1__^(*c*_*n*_)^} and *I*_2_ = {*I*_*A*_2__^(*c*_0_)^,…, *I*_*A*_2__^(*c*_*n*_)^} represent their caching strategy sets. The utility functions can be rewritten as *U*_*A*_1__(*I*_1_, *I*_2_) and *U*_*A*_2__(*I*_1_, *I*_2_), and both their values depend on the two strategy set *I*_1_ and *I*_2_. From the formulas (3) and (4) we see that *U*_*A*_1__(*I*_1_, *I*_2_) is convex and continuous on the strategy sets *I*_1_ of ISP *A*_1_ and so is the function *U*_*A*_2__(*I*_1_, *I*_2_) on *I*_2_ of ISP *A*_2_. Since the value of any caching strategy *I*_*A*_*i*__^(*c*_*j*_)^ belongs to the 2-element field {0,1}, the caching strategic vector spaces *I*_1_ and *I*_2_ are both closed spaces. ISPs *A*_1_ and *A*_2_ independently decide their own strategies *I*_1_ and *I*_2_ to minimize their respective utility functions. Hence, if function *U*_*A*_1__(*I*_1_, *I*_2_) gets the minimal value at a solution *I*_1_* and *U*_*A*_2__(*I*_1_, *I*_2_) gets the minimal value at *I*_2_*, we have the inequations *U*_*A*_1__(*I*_1_*, *I*_2_*) ≤ *U*_*A*_1__(*I*_1_, *I*_2_*) and *U*_*A*_2__(*I*_1_*, *I*_2_*) ≤ *U*_*A*_2__(*I*_1_*, *I*_2_). When ISP *A*_1_ chooses *I*_1_* and ISP *A*_2_ chooses *I*_2_*, they will not deviate from this equilibrium solution, because they cannot gain more profit with other strategies. Hence, (*I*_1_*, *I*_2_*) is a Nash equilibrium point.

In the tit-for-tat case, the sharing strategies *X*_*ik*_^(*c*_*j*_)^ ∈ [0,1] of ISP *A*_*i*_ also generate a closed space. So, following a similar way, the existence of equilibria in tit-for-tat and altruism can be proved. As for the cooperation case, Proposition 1 has proved that a stable coalition exists in the cooperative game.

### 5.2. Dynamic Convergence

After building the peering, an ISP commits itself to respond to content requests from intradomain or from its peers. During a period of time, the peering ISPs can dynamically converge to equilibrium of their peering strategic game. We can detail the dynamic convergence by an example of the egoistic game between two ISPs. In this noncooperative game, ISPs *A*_1_ and *A*_2_ take several rounds to converge to a strategic equilibrium, that is, the optimal content delivery cost which they will not deviate from by choosing other caching or sharing strategies. In each round, according to the strategic decision of ISP *A*_2_, ISP *A*_1_ will react and adjust with an optimal strategy (of this round) by updating its cache. Conversely, ISP *A*_2_ will react to strategic change of ISP *A*_1_ in the next round and make an optimal adjustment. Although this noncooperative game alternates the two ISPs to make strategic change, it will converge to the equilibrium after several rounds, since the strategies of both arrive at the optimal one and do not need to change again. This dynamic convergence is also the base of algorithm design for simulating the game process.

### 5.3. Divergence from Social Optimum

Evaluating the equilibrium of a game may go separate ways since we view the optimal solution of the game from different perspectives. Now we consider a peering strategic game with multiple ISPs as participants. From the point of view of every single ISP, the equilibrium solution is optimal because it cannot gain a lower cost of content delivery by choosing other peering strategies. However, from a global point of view, a highly evaluated cost at the equilibrium of a game should not diverge too much from the social optimum, which we have defined in Section 4. To study the game's divergence from the social optimum, we use the price of anarchy (PoA) [15] to measure the inefficiency of the decentralized optimization. Suppose that *n* ISPs participate in the cost optimization. Let *I*_*i*_ = {*I*_*A*_*i*__^(*c*_0_)^,…, *I*_*A*_*i*__^(*c*_*l*_)^} represent the caching strategy of ISP *A*_*i*_, and *Χ*_*i*_ = {*X*_*ik*_^(*c*_0_)^,…, *X*_*ik*_^(*c*_*l*_)^} represent the sharing strategy. For a noncooperative game, *U*_*A*_*i*__(*I*_1_,…, *I*_*n*_, *Χ*_1_,…, *Χ*_*n*_) denotes the cost of ISP *A*_*i*_. For the global optimization, *U*_Global_ denotes the total cost of *n* ISPs at the global optimum. PoA is expressed as follows:

(14) $$\begin{array}{c} \text{PoA}=\underset{i,j}{\sup}\frac{\sum_{c_{j}}\sum_{i}U_{A_{i}}\left(I_{1},\ldots ,I_{n},{Χ}_{1},\ldots ,{Χ}_{n}\right)}{U_{\text{Global}}}. \end{array}$$

In the numerical results, PoA of the four peering games will be given.

## 6. Numerical Results

**Figure 2:**
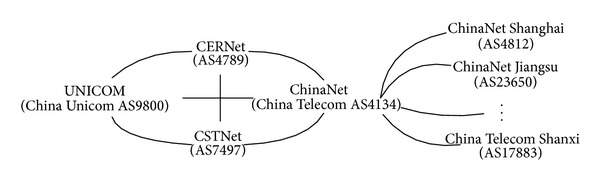
Connection of some large networks in the Chinese Internet.

To evaluate performance of interdependent ISPs in the proposed peering strategic game models, we simulate the games on a toy system whose AS-level peering topology represents the interconnection between some large networks of the Chinaese Internet [16] as shown in Figure 2. The topology has totally 32 autonomous systems (ASs) including two academic networks, CERNet and CSTNet, two commercial networks, ChinaNet and UNICOM, and 28 local ISPs of provincial and municipal access networks connected to the ChinaNet Backbone. Based on this topology, we study global-optimum-divergence and communication cost changed with the caching and sharing strategies of ISPs in different game models and explore the proper peering strategy to balance intra-ISP content demand and inter-ISP relation for cost saving. According to the scale of networks (ChinaNet has 100 million broadband Internet access customers [17]; UNICOM has 30 million broadband subscribers [18]; CERNet has 20 million end users [19]; CSTNet has 1 million end users [20]), we assign ChinaNet a cache capacity of 1, UNICOM a capacity of 0.3, CERNet a capacity of 0.2, and CSTNet a capacity of 0.01. We also assign each of the 28 access ISPs a capacity of 0.005.

### 6.1. Price of Anarchy

**Figure 3:**
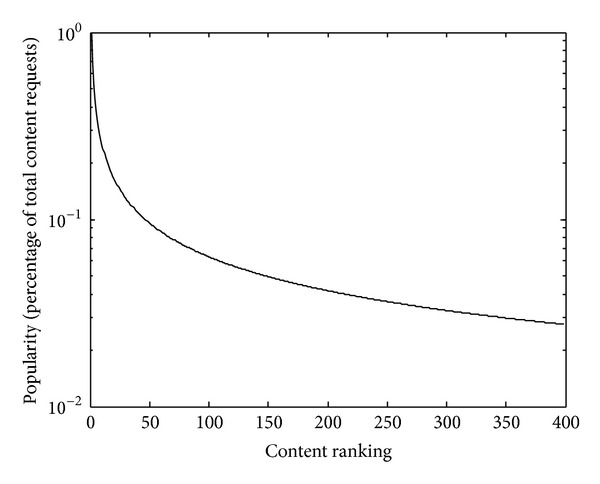
Content popularity in a Zipf-like distribution.

Figure 3 shows the popularity of content that follows Zipf-like distribution in [12] with a default parameter *α* = 0.6. Let *p*_*A*_*i*__^(*c*_*j*_)^ denote the percentage of requests for *c*_*j*_ within ISP *A*_*i*_. For each ISP *A*_*i*_, we select *p*_*A*_*i*__^(*c*_*j*_)^ from the ranked content items in Figure 3 and let the average content popularity of selected content items vary in the range 0, 1. Then we study the PoA change with content popularity.

**Figure 4:**
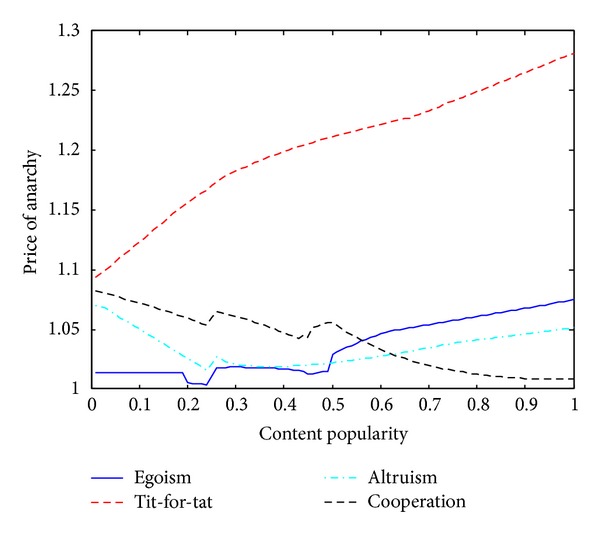
Price of anarchy versus content popularity.

We deploy the four peering strategic games on the topology of 4 ASs (CERNet, CSTNet, ChinaNet, and UNICOM) in Figure 2 and let U* denote the total content delivery cost of the 4 ASs at the equilibrium of each game. *U*_Global_ denotes the total cost at the global optimum solution as formula (13). Price of anarchy (PoA) [15] is expressed as PoA = U*/*U*_Global_. Value of PoA indicates how far a game's equilibrium solution deviates from the social optimum solution. We evaluate PoA because we want to know whether a near-social-optimal peering exists in the four games.  Figure 4 shows the four games' PoA values changed with popularity of our selected content sets. The four games' PoA values vary in a range from nearly 1 to 1.28. The tit-for-tat model's PoA has a varying range length up to nearly 0.2, while the varying range lengths in the other three models are all less than 0.1. Both egoism and tit-for-tat have a broadly increasing figure patterns. Yet, tit-for-tat has a much larger PoA than egoism, since tit-for-tat makes a partial rather than total content contribution and limits the sharing relationship between ISPs. When the content popularity is less than 0.5, the egoism case is the near-optimal peering model. When there is too much content demand with popularity more than 0.6, the relatively altruistic peering cases (altruism and cooperation) outperform the relatively selfish case (egoism and Tit-for-tat) with respect to the divergence from global optimum. The cooperation case shows a roughly decreasing figure pattern and has near-optimal PoA values in high popularity range.

### 6.2. Content Delivery Cost

**Figure 5:**
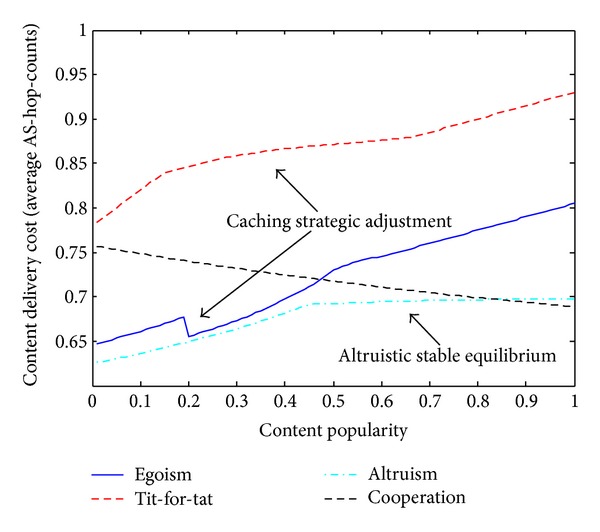
Content delivery cost versus content popularity.

We use average AS-hop-count as the metric of the communication cost to receive content within the AS or from other ASs. As shown in Figure 5, costs of all the four models are less than one AS-hop-count due to proper content-caching strategy decided by a rational ISP to cache more locally popular content and decrease times of cross-AS communication for content delivery cost saving. When content popularity increases up to very high values, caching strategy encounters the bottleneck. It is difficult for an ISP to serve its clients independently. Increasing frequency of cross-AS communication results in increasing cost for the three peering relationships except cooperation. Although having relative high cost in low popularity range, cooperation shows a decreasing figure pattern and outperforms other models in very high popularity range. Egoism and tit-for-tat show caching strategic adjustment in their figures. Through caching more and more intradomain popular contents, egoistic peering ISPs lower the cost at the popularity range from 0.18 to 0.2, while tit-for-tat ISPs alleviate the sharply increasing trend of cost figure with a smaller slope during popularity range from 0.2 to 0.7. Additionally, at popularity range from 0.45 to 1, altruism model shows a stable equilibrium cost values which nearly do not increase with content popularity. Such good properties are instructive for ISPs to select proper peering strategy in different situations of content popularity.

### 6.3. Impact of ISPs Number

**Figure 6:**
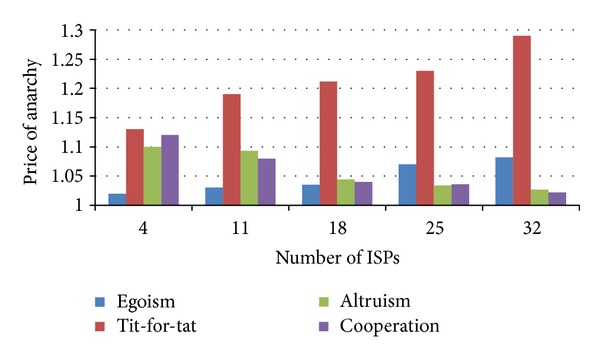
Price of anarchy versus ISP number.

**Figure 7:**
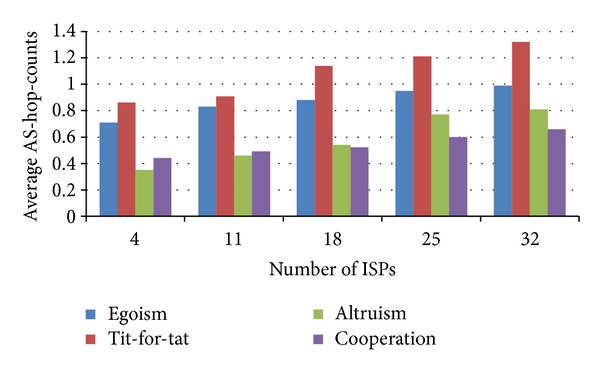
Content delivery cost versus ISP number.

The number of ISPs also exerts an impact on the performance of the four games. We fix the values of content popularity and let the number of ISPs vary from 4 to all 32 ASs.  Figure 6 shows the values of PoA change with ISPs number. The PoA of comparatively egoistic types (egoism and tit-for-tat) increases with the number of ISPs, yet the PoA of comparatively altruistic types (altruism and cooperation) decreases with the number of ISPs. According to formulas (3), (5), (8), and (12) and Proposition 1, the changes in Figure 6 is due to the advantage of coalitional and cooperative games that are well adapted to a large scale of AS-level topology with a large number of peering ISPs.  Figure 7 shows the content delivery costs change with ISPs number. The average AS-hop-counts of all the four types increase with the number of ISPs. This is so because when the ISP number is relatively small, an ISP has few choices, caches most content items by itself, and accordingly lowers the times to do the cross-ISP delivery; when the ISP number becomes large, an ISP has increasing possibility to receive content from its adjacent ISPs or other ISPs.

### 6.4. Sharing Strategy in Tit-for-Tat

Sharing strategies in the peering relationship also have influence on the ISP's content delivery cost. In this subsection, we study two types of sharing function *f*(*x*) in formula (5).

Considering the peering relationships between *n* ISPs, we formulate a sharing function as follows:

(15) $$\begin{array}{c} f_{j}\left(X_{ij}^{\left(c_{l}\right)}\right)=\frac{p_{A_{j}}^{\left(c_{l}\right)}\cdot X_{ij}^{\left(c_{l}\right)}}{\sum_{k=1}^{n}p_{A_{k}}^{\left(c_{l}\right)}\cdot X_{ik}^{\left(c_{l}\right)}}. \end{array}$$

This formula denotes that peering ISP *A*_*j*_ will decide its sharing strategy according to the proportion of the contribution *p*_*A*_*j*__^(*c*_*l*_)^ · *X*_*ij*_^(*c*_*l*_)^ to ISP *A*_*i*_'s total contribution to all peers. The cost utility function of ISP *A*_*i*_ can be expressed as follows:

(16) UAi=∑clpAi(cl)·(D0·IAi(cl)        +∑Aj≠AiD1·fj(Xij(cl))·(1−IAi(cl))·IAj(cl)).

**Figure 8:**
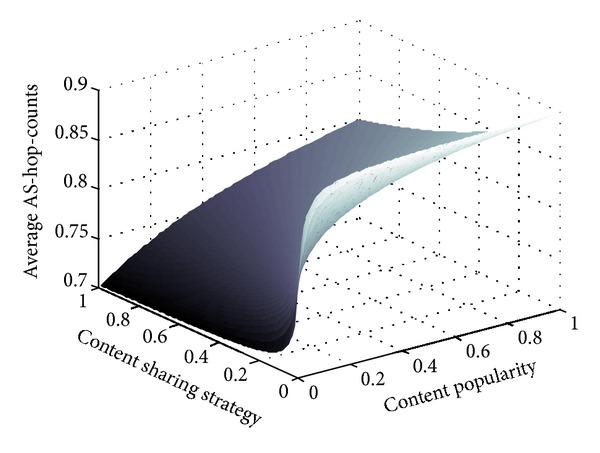
Content delivery cost in tit-for-tat peering.

To optimize the utility, an ISP has to balance its intradomain content demand and the peering relationship with other ISPs.  Figure 8 shows a peering ISP's content-receiving cost change with both content popularity and content sharing strategies in the tit-for-tat game model. The average AS-hop-counts still broadly increase with content popularity. When content popularity is very low (from 0 to 0.05), sharing strategy varying from 0 to 1 does not affect the cost remarkably. Yet, when content popularity increases up to 1, the cost changes significantly from 0.9 to 0.8 across the sharing strategic varying range from 0 to 1. Hence, the peering ISP should decide proper sharing strategy in different situations. With great demand for content, peering ISPs should contribute more to each other for reciprocal content delivery.

Another type of the peering functions are expressed as *f*_*i*_(*X*_*ji*_^(*c*_*l*_)^) = *X*_*ij*_^(*c*_*l*_)^ and *f*_*j*_(*X*_*ij*_^(*c*_*l*_)^) = *X*_*ji*_^(*c*_*l*_)^. Function *f*_*i*_(*x*) is the inverse function of *f*_*j*_(*x*) and vice versa. Such mutually inverse property can instruct peering ISPs to design their sharing strategy as a function symmetric to the line *y* = *x*. The advantage of this design lies in that the two peering ISPs will have the same sharing function and monotonicity so that one ISP can precisely predict the sharing strategy of the other one.

## 7. Discussion

There are other highly involving issues that we ought to pay attention to.

First, the cost evaluation in our numerical analysis uses the average value of equilibrium in games between couples of peering ISPs. The actual interdependence and interaction processes among the involved ISPs are more complex than the simple combination of some two-person games. Yet, our models are the elementary games that are able to generate the comprehensive interrelationships, thus showing similar properties as the actually complex interactions. In addition, since distinct patterns about the cooperative willingness and peer's independent decisions are embodied in the game models, the numerical results will also reflect the cost trends of content delivery in distinct peering relationships. These results are instructive for an ISP's peering strategy choice.

Second, from a practical point of view, ISPs still base their peering relations on the fundament of peering agreements and data exchange protocols between networks. With regard to the current Internet policies, ISPs rely on two basic types of contracts (peering and transit) for exchanging traffic. Compared with traditional interconnection agreements among ISPs, peering for content needs not only the agreements to route traffic, but also a series of distributed networking protocols deployed on the boundary nodes of ISPs' networks to efficiently exchange content between the mutually recognized ISP peers. This should be elaborated in the new networking design.

Third, the Internet is experiencing the transition from a transit hierarchy to a peering mesh and becoming flat [10, 11]. It means that there are more and more peering ISPs at the same tier level. This evolution is good for wide adoption of content-based peering in the future Internet architecture. From our numerical analysis about the values of PoA and average AS-hop-count, we see that the tit-for-tat case, whose interaction between peers is similar to the transit hierarchy, does not have a good performance in the evaluation. This may prompt ISPs to adapt themselves with more cooperatively peering strategies, which will motivate sharing of content-based ISP peers.

Finally, networks in the future Internet will arrive at a high level of intelligence, which means that an ISP's network will no longer be limited to the “stupid” data carrier and will actively involve content management for fulfilling QoS [21, 22]. Such intelligence is always related to the optimization mechanism with which the network can decide the optimal policy for content delivery. Multiple intelligent networks are supposed to use cooperative way to a “win-win” situation. Our proposed peering strategic game models can be applied for finding the optimal equilibrium solutions.

## 8. Conclusion

In this paper we propose the concept of peering for content and four game models for peering ISPs to decide content caching and sharing strategies and minimize communication cost of content delivery in content centric Internet. In different games, peering ISPs are characterized by distinct degree of cooperative willingness from egoism to altruism and are interconnected as profit-individuals or profit-coalition. Based on the AS-level topology of part of the Chinese Internet, we simulate the four games to evaluate the performance of price of anarchy value and content delivery cost resulted from ISPs' peering strategy selection. Peering relationships in our proposed games show some good properties (e.g., near-social-optimal PoA, caching strategic adjustment for cost-saving, and sharing strategic balance between intracontent demand and inter-ISP relation), which are instructive for ISPs in both the current and the future content-centric Internet.
